# Prediction of Metastasis-Free Survival in Patients with Localized Prostate Adenocarcinoma Using Delta Radiomics from Pre-Treatment PSMA-PET/CT Scans and Dosiomics

**DOI:** 10.3390/cancers18040677

**Published:** 2026-02-19

**Authors:** Apurva Singh, William Silva Mendes, Sang-Bo Oh, Ozan Cem Guler, Aysenur Elmali, Birhan Demirhan, Amit Sawant, Phuoc Tran, Cem Onal, Lei Ren

**Affiliations:** 1 Department of Radiation Oncology, University of Maryland School of Medicine, Baltimore, MD 21201, USA; apurva.singh@som.umaryland.edu (A.S.); wsilvamendes@som.umaryland.edu (W.S.M.); sangboimda@hanmail.net (S.-B.O.); asawant@som.umaryland.edu (A.S.); pttran6@mdanderson.org (P.T.); 2 Department of Internal Medicine, Division of Medical Oncology and Hematology, Pusan National University Yangsan Hospital, Pusan National University School of Medicine, Yangsan 50612, Republic of Korea; 3 Adana Dr Turgut Noyan Research and Treatment Center, Department of Radiation Oncology, Baskent University Faculty of Medicine, Adana 01250, Turkey; ocguler@baskent.edu.tr (O.C.G.); birhandemirhan@baskent.edu.tr (B.D.); 4 Department of Radiation Oncology, Baskent University Faculty of Medicine, Ankara 06490, Turkey; aysenurelmali@baskent.edu.tr; 5 Department of Genitourinary Radiation Oncology, The University of Texas MD Anderson Cancer Center, Houston, TX 77030, USA

**Keywords:** prostate cancer, PSMA-PET/CT, delta radiomics, dosiomics, prognostic modeling

## Abstract

Purpose: To develop prognostic models integrating delta radiomics from prostate-specific membrane antigen positron emission tomography/computed tomography (PSMA-PET/CT) and dosiomics with clinical variables to predict metastasis-free survival (MFS) in patients with localized prostate adenocarcinoma treated with androgen deprivation therapy and external-beam radiotherapy. Materials/Methods: Delta-radiomics analysis included 43 patients. Radiomics features were extracted from the primary tumor on pre- and post-treatment PSMA-PET/CT, and delta features were calculated as relative changes. Eight high-variance features were selected and combined with clinical variables (age, Gleason score, initial PSA, and a binary variable, indicating the occurrence of PSA relapse). Data was split 70:30 with training-set imbalance correction. Predictors that were significant in univariate Cox regression (*p* < 0.05) were entered into multivariate Cox models, and five-year MFS was classified using a quadratic support vector machine. Dosiomics analysis included 48 patients. Dosiomics features were extracted from the planning target volume receiving 86 Gy and combined with pre-treatment radiomics and clinical variables using the same framework. Results: For delta radiomics, Model 1 (delta radiomics + pre-treatment radiomics + clinical) achieved the best performance (test c-score 0.58; AUC 0.70), exceeding Model 2 (pre-treatment radiomics + clinical; c-score 0.56; AUC 0.65) and Model 3 (clinical only; c-score 0.51; AUC 0.56). For dosiomics, Model 1 showed the highest performance (test c-score 0.56; AUC 0.67) compared with Model 2 (c-score 0.55; AUC 0.62) and Model 3 (c-score 0.50; AUC 0.54). Conclusions: Integrating delta radiomics or dosiomics with pre-treatment imaging and clinical variables improves MFS prediction and supports their role as non-invasive biomarkers for individualized radiotherapy in localized prostate cancer.

## Simple Summary

This study develops prognostic models combining delta radiomics from PSMA-PET/CT, dosiomics, and clinical variables to predict metastasis-free survival (MFS) in patients with localized prostate adenocarcinoma treated with androgen deprivation therapy and external-beam radiotherapy. Delta radiomics features were computed from primary tumor volumes on pre- and post-treatment PSMA-PET/CT scans, while dosiomics features were derived from the intra-prostatic lesion receiving 86 Gy in the planning CT scans. Selected high-variance radiomics and dosiomics features were integrated with clinical factors, including age, Gleason score, baseline PSA, and PSA relapse. Data were split into training and testing cohorts with imbalance correction, and prognostic factors were evaluated using Cox regression and five-year MFS classification. Models incorporating delta radiomics or dosiomics with pre-treatment imaging and clinical variables consistently outperformed clinical factor-only models, achieving higher concordance indices and AUC values. These findings demonstrate that integrating biomarkers capturing temporal radiomics and spatial dose heterogeneity with clinical data improves prognostic accuracy and supports the use of radiomics and dosiomics as non-invasive tools for personalized radiotherapy planning in localized prostate cancer.

## 1. Introduction

Prostate-specific membrane antigen positron emission tomography/computed tomography (PSMA-PET/CT) has become the preferred imaging modality for staging and treatment planning in prostate cancer (PCa), offering superior sensitivity for nodal and distant metastases (DMs) compared with conventional imaging [1,2]. Beyond staging, PSMA-PET/CT provides quantitative information that may reflect tumor biology, yet its potential for outcome prediction remains underexplored [3,4,5].

Radiomics enables the extraction of high-dimensional quantitative features from medical images, converting voxel-level heterogeneity into measurable biomarkers [6]. In PCa, radiomic signatures derived from multiparametric magnetic resonance imaging (mpMRI) or PSMA PET/CT have shown promise in predicting biochemical recurrence and metastasis [4,5,7]. However, static pre-treatment imaging may not fully capture the dynamic biological response of the tumor and surrounding tissue during therapy.

Delta radiomics, which evaluates changes in image-derived features between serial imaging time points, offers a means to assess treatment-related biological adaptation. Recent studies in lung, head and neck, and cervical cancers have demonstrated that delta radiomic features outperform static radiomics in predicting response and survival outcomes [8,9,10,11]. Nevertheless, data on delta radiomics in PCa are scarce, and integration with PSMA-PET/CT remains largely unexplored.

Parallel to radiomics, dosiomics, the extraction of spatial dose distribution features from radiotherapy (RT) plans, has emerged as a complementary approach to characterize delivered radiation patterns beyond conventional dose volume metrics [12]. Dosiomics can quantify spatial heterogeneity in dose deposition within the target and surrounding organs at risk, potentially correlating with tumor control or toxicity risk [13]. Despite its relevance, few studies have combined dosiomics with imaging-based biomarkers to predict oncologic outcomes [14,15,16,17].

In localized PCa, treatment personalization remains a clinical priority. While PSMA PET/CT has markedly improved staging accuracy, reliable imaging-based predictors of long-term metastasis-free survival (MFS) after definitive RT are still lacking [5,7]. Integrating delta radiomics, which captures temporal biological changes during treatment, with dosiomics, representing spatial radiation effects within the target and surrounding tissues, may provide a more comprehensive framework for outcome prediction than either approach alone [15,16,17].

Therefore, the present study aimed to develop and validate prognostic models for MFS in patients with localized PCa using pre-treatment PSMA-PET/CT radiomics, delta radiomics, and dosiomics. We hypothesized that combining temporal and spatial imaging-derived features with established clinical variables would improve individualized risk stratification and enable early identification of patients at higher risk of metastatic progression, supporting more adaptive and personalized treatment strategies.

## 2. Materials and Methods

### 2.1. Patient Cohort

This retrospective study included 134 patients with localized PCa treated at Başkent University between 2014 and 2023. Institutional Review Board approval was obtained (HP-00100523), and all procedures complied with the Declaration of Helsinki under a waiver of informed consent.

Eligible patients had histologically confirmed prostate adenocarcinoma and underwent staging with PSMA-PET/CT. All were treated with external-beam RT (EBRT) with or without androgen deprivation therapy (ADT). Patients who had prior radical prostatectomy, neoadjuvant chemotherapy, prostate-only RT, or evidence of DM were excluded.

Definitive RT was delivered using intensity-modulated or volumetric-modulated arc therapy. The prescribed prostate dose ranged from 76 to 78 Gy, and a simultaneous integrated boost (SIB) to the intraprostatic lesion up to 86 Gy was delivered when feasible [18,19]. The pelvic lymphatic field received 46–54 Gy without an additional nodal boost. ADT consisted of an LHRH agonist alone or in combination with bicalutamide; no patient received next-generation androgen receptor-targeted agents such as enzalutamide, abiraterone, darolutamide, or apalutamide.

**Figure 1:**
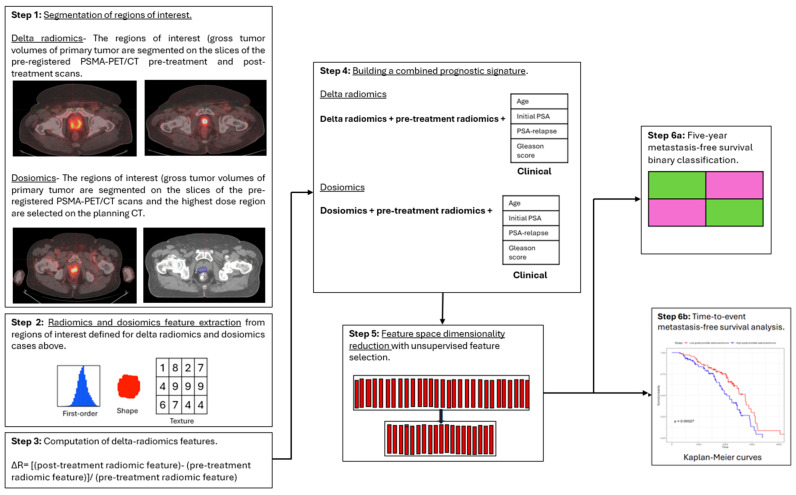
Workflow illustrating the methodological pipeline for delta-radiomics and dosiomics analyses. Step 1: Segmentation of regions of interest (ROIs). Step 2: Extraction of radiomics and dosiomics features. Step 3: Computation of delta radiomics features. Step 4–5: Integration with clinical variables, dimensionality reduction. Step 6a–6b: Evaluation.

Two analytic subsets were defined: (i) a delta-radiomics cohort of 43 patients who underwent both pre- and post-treatment PSMA-PET/CT scans (mean interval, 10 months), and (ii) a dosiomics cohort of 48 patients with available planning CT data and three-dimensional dose distributions (Figure 1).

### 2.2. Overall Workflow

A brief explanation of the workflow illustrated in Figure 1 is as follows:

Step 1: Segmentation of regions of interest (ROIs) on pre- and post-treatment PSMA-PET/CT scans for delta radiomics and on pre-treatment PSMA-PET/CT and planning CT scans for dosiomics. Step 2: Extraction of radiomics and dosiomics features (first-order, shape, and texture). Step 3: Computation of delta radiomics features (ΔR = [post- − pre-treatment]/pre-treatment value). Step 4–5: Integration with clinical variables (age, initial PSA, PSA relapse, and Gleason score) and unsupervised feature selection for dimensionality reduction. Step 6a–6b: Evaluation through five-year metastasis-free survival (MFS) binary classification and time-to-event metastasis-free survival analysis.

### 2.3. Imaging Acquisition and Segmentation

All PSMA-PET/CT examinations were acquired using a Discovery STE 8 scanner (GE Healthcare, Milwaukee, WI, USA). PET images were reconstructed with matrices of 192 × 192 × 567 or 128 × 128 × 367 voxels and voxel spacing of approximately 3.7–5.5 × 3.3 mm^3^. CT images were obtained with 512 × 512 × 567 matrices, voxel spacing of 1.4 × 3.3 mm^3^, and a tube voltage of 140 kVp.

**Figure 2:**
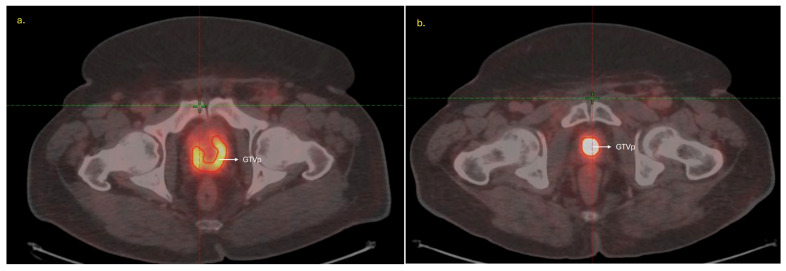
Representative PSMA-PET/CT images showing the gross tumor volume of the prostate (GTVp) delineated on (**a**) pre-treatment and (**b**) post-treatment registered scans in a patient included in the delta-radiomics analysis.

**Figure 3:**
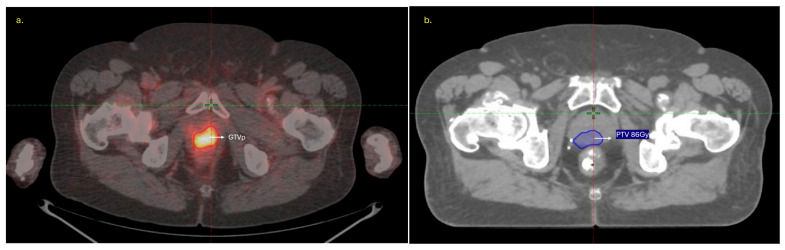
Representative images for the dosiomics analysis showing (**a**) the GTVp contoured on pre-treatment PSMA-PET/CT and (**b**) the corresponding intra-prostatic region (planning target volume (PTV) receiving 86 Gy) on the planning CT.

Gross tumor volumes of the primary prostate lesion (GTVp) were delineated on co- registered PSMA-PET and CT images by two radiation oncologists in consensus. PET images were resampled to match CT voxel dimensions before feature extraction. In the delta-radiomics subset, GTVp was segmented on both pre- and post-treatment scans, whereas in the dosiomics subset, GTVp was contoured on the pre-treatment PSMA-PET/CT, and the planning target volume (PTV) receiving 86 Gy on the planning CT (intra-prostatic lesion) served as the region of interest for dosiomics analysis. Representative segmentations for both analyses are shown in Figure 2 (delta radiomics) and Figure 3 (dosiomics).

### 2.4. Radiomics and Dosiomics Feature Extraction

Radiomics and dosiomics features were extracted using PyRadiomics (3D Slicer v5.2) (Supplementary Table S1) [20]. For each region of interest, 107 quantitative features were derived, including shape descriptors, first-order statistics, and second- and higher-order textural matrices (GLCM, GLRLM, GLSZM, GLDM, and NGTDM). All features were standardized via Z-score normalization.

For delta radiomics, features were extracted from both pre- and post-treatment PET/CT scans, and relative change was calculated for each feature as:

ΔR = (R post − R pre)/R pre

Dosiomics features were extracted from the dose-distribution map corresponding to the PTV receiving 86 Gy (intra-prostatic lesion). These features quantified spatial dose heterogeneity and were normalized using the same Z-scoring procedure.

Four clinical parameters were included across all models based on prior evidence of prognostic relevance: age, ISUP grade (Gleason score), baseline PSA value, and PSA-relapse category (biochemical recurrence vs. none).

### 2.5. Feature Selection and Model Construction

Separate prognostic models were developed for the delta-radiomics and dosiomics datasets. For each analysis, three models were generated:

- Model 1 combined delta radiomics or dosiomics with pre-treatment PSMA-PET/CT radiomics and clinical factors;
- Model 2 integrated pre-treatment PSMA-PET/CT radiomics with clinical factors;
- Model 3 included clinical factors alone.

To reduce dimensionality, variance-thresholding identified the most informative predictors; eight for delta-radiomics and seven for dosiomics analyses. The list of the top eight features for delta-radiomics and the top seven features for dosiomics analyses has been included in Supplementary Table S2. Principal-component analysis was initially evaluated but yielded inferior predictive performance; therefore, variance-based feature selection was used for all final models. The performance of the models built using the PCs approach has been included in Supplementary Tables S3 and S4 (delta-radiomics analysis) and Tables S5 and S6 (dosiomics analysis) for reference.

### 2.6. Model Training and Validation

#### 2.6.1. Time-to-Event Metastasis-Free Survival Analysis

The primary endpoint was MFS, defined as the time from completion of RT to radiologically confirmed metastasis or last follow-up. Due to the limited number of metastatic events (10/43 in the delta-radiomics cohort and 12/48 in the dosiomics cohort), data were randomly divided into 70:30 training and testing subsets. The Synthetic Minority Over-Sampling Technique (SMOTE) was applied to correct class imbalance in the training dataset.

Univariate Cox regression (*p* < 0.05) identified candidate predictors, which were entered into a multivariate Cox proportional hazards model trained on the balanced dataset and validated on the independent test set. Model discrimination was quantified using the Harrell concordance index (c-index).

#### 2.6.2. Five-Year MFS Classification

For binary outcome assessment, a Quadratic Support Vector Machine (SVM) classifier was applied with five-fold cross-validation to predict 5-year MFS. Performance metrics included area under the receiver operating characteristic curve (AUC), sensitivity, and specificity. Kaplan–Meier curves were plotted to visualize risk stratification by model-derived groups, with differences assessed using the log-rank test.

### 2.7. Statistical Software

All statistical procedures were performed in Python 3.7 (Anaconda distribution) for feature extraction, imbalance correction, and Cox modeling; MATLAB R2022b for machine learning classification; and R v4.2.2 for survival analysis. All statistical tests were two-sided, with *p* < 0.05 considered statistically significant.

## 3. Results

In the delta-radiomics cohort, models incorporating imaging-derived delta-radiomics features demonstrated superior predictive performance compared with those based solely on pre-treatment radiomics and clinical variables (Table 1). The integrated model combining delta features, pre-treatment radiomics, and clinical parameters (Model 1_delta) achieved the highest concordance index (c-index = 0.64; 95% CI, 0.58–0.65 in the training set and 0.58; 95% CI, 0.53–0.59 in the test set). This was followed by Model 2 (pre-treatment radiomics + clinical variables; c-index = 0.62 [0.57–0.63] in training and 0.56 [0.52–0.58] in testing). The clinical factor-only model (Model 3) demonstrated the lowest discriminative ability (c-index = 0.57 [0.51–0.58] in training and 0.51 [0.50–0.55] in testing). These findings indicate that the inclusion of delta-radiomics features derived from PSMA-PET/CT improves prognostic modeling of MFS compared with pre-treatment radiomics and clinical factors alone.

Similarly, in the dosiomics cohort, models that incorporated dose features provided higher predictive accuracy than those relying solely on pre-treatment radiomics and clinical variables. The comprehensive model integrating dosiomics, pre-treatment radiomics, and clinical parameters (Model 1_dose) achieved the best performance (c-index = 0.60 [0.56–0.61] in training and 0.56 [0.54–0.59] in testing), followed by Model 2 (c-index = 0.58 [0.55–0.60] in training and 0.55 [0.52–0.57] in testing) (Table 1). The addition of dosiomics features to conventional clinical and imaging variables, therefore, enhanced the ability to predict MFS.

Using the Quadratic Support Vector Machine (SVM) classifier, the delta-radiomics model combining delta, pre-treatment, and clinical features (Model 1_delta) achieved the highest classification accuracy (AUC = 0.70, sensitivity = 65.1%, specificity = 71.2% on the test set). Both Model 1_delta and Model 2 outperformed the clinical factor-only model (Model 3) (Table 2).

Regarding the dosiomics study, the combined model incorporating dosiomics, pre- treatment radiomics, and clinical variables (Model 1_dose) achieved the highest classification accuracy, with both Model 1_dose and Model 2 outperforming the clinical factor-only model (Model 3). Detailed Cox regression and five-year MFS classification results for principal component-based models are summarized in Supplementary Tables S2–S5.

**Figure 4:**
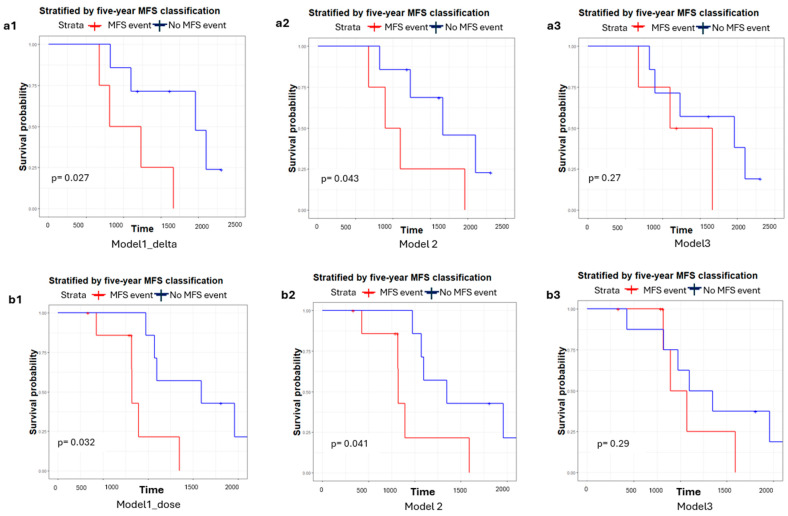
Kaplan–Meier survival curves stratified by five-year metastasis-free survival (MFS) classification (MFS event vs. no event). Panels (**a1**–**a3**) show the delta-radiomics models: (**a1**) Model 1_delta = delta radiomics + pre-treatment radiomics + clinical; (**a2**) Model 2 = pre-treatment radiomics + clinical; (**a3**) Model 3 = clinical only. Panels (**b1**–**b3**) show the dosiomics models: (**b1**) Model 1_dose = dosiomics + pre-treatment radiomics + clinical; (**b2**) Model 2 = pre-treatment radiomics + clinical; (**b3**) Model 3 = clinical only.

Kaplan–Meier curves of patients stratified by five-year MFS predictions were plotted in Figure 4 to evaluate their survival differences to determine the efficacy of stratification. The Model1_delta integrating delta-radiomics features (Figure 4a1) achieved statistical significance in separation with a log-rank *p* = 0.027, whereas Model 2 approached statistical significance (*p* = 0.043). In contrast, Model 3 failed to yield significant stratification (*p* = 0.27).

In the dosiomics study, the Kaplan–Meier (KM) curves stratified by the five-year MFS classification are shown in Figure 4b1–b3. The Model1_dose incorporating dosiomics information showed a statistically significant separation between patients predicted to experience an MFS event versus those predicted not to (log-rank *p* = 0.032 in Figure 4b1). A near-significant separation was observed for Model 2 (*p* = 0.041), whereas the clinical factor-only model (Model 3) failed to achieve significance (*p* = 0.29). These findings confirm that the integration of dosiomics features with pre-treatment PSMA-PET/CT radiomics enhances risk stratification for MFS.

## 4. Discussion

Prostate cancer is one of the most common malignancies in men. Advances in the field of precision medicine for oncology have often stressed the importance of developing machine learning-based decision support tools in the identification of suspected malignant nodules in a non-invasive manner and their integration into routine surveillance workflows. For instance, in the recent study by Telecan et al., the authors developed a machine learning tool from multi-parametric T2-weighted MRI images to differentiate benign and malignant lesions, and classify each nodule into corresponding ISUP grades prior to prostate biopsy [21]. PSMA-PET/CT has rapidly evolved as an indispensable imaging modality in PCa, offering superior lesion detection and biologically meaningful quantification of disease burden. Beyond diagnostic use, PSMA-PET-derived quantitative and radiomics features have shown prognostic potential, particularly in oligometastatic or recurrent settings where metabolic changes predict treatment response and survival [22,23,24]. PET/CT has been shown to contribute metabolic information to anatomical imaging information; radiomics features derived from texture matrices, combined with uptake information, have strong potential for outcome prediction and treatment response monitoring [25]. However, its role in predicting long-term outcomes in localized PCa remains underexplored, despite growing evidence that PSMA expression and uptake dynamics reflect tumor aggressiveness and microenvironmental heterogeneity [26,27].

Building upon our prior work in a cohort of 134 patients, which demonstrated that combining primary-tumor, nodal, and peritumoral ring radiomics with clinical factors enhances MFS prediction [5], the present study advances this framework by incorporating delta-radiomics and dosiomics biomarkers. These complementary dimensions capture distinct biological and physical phenomena. Delta radiomics quantifies temporal changes in intratumoral PSMA uptake and CT-based texture following RT, potentially reflecting early tumor regression, PSMA downregulation, or microvascular normalization [15,16,27]. Dosiomics characterizes the spatial complexity of dose delivery within high-dose regions, which may correlate with heterogeneous sub-voxel radiosensitivity and residual clonogenic potential [12,28,29,30,31].

Our findings indicate that integrating delta-radiomics or dosiomics features with pre-treatment PSMA-PET/CT radiomics and clinical parameters yields superior prognostic performance for MFS compared with clinical or baseline radiomics models alone. These results align with studies in other tumor sites where delta radiomics and dosiomics improved survival prediction and treatment response modeling [12,15,16,30,31]. It is imperative to discuss here that although the improvement in the performance described above is moderate, the results suggest that longitudinal imaging-derived descriptors of biological adaptation and spatial dose heterogeneity capture complementary prognostic information not reflected in static imaging or clinical features. Importantly, these results should be interpreted as promising but preliminary. The added value of delta radiomics and dosiomics requires confirmation in larger, multi-institutional, external validation cohorts, prospectively collected cohorts with standardized acquisition and reconstruction protocols, to assess model robustness, generalizability, and reproducibility across heterogeneous imaging platforms and clinical settings. Such validation efforts are critical for translating these exploratory findings into clinical decision-support tools.

From a clinical standpoint, these approaches have meaningful translational implications. Several PSMA-PET/CT radiomic features, particularly texture- and heterogeneity-based metrics, may plausibly reflect underlying tumor characteristics such as spatial variability in PSMA expression, cellular density, and microenvironmental complexity, which are associated with higher ISUP grade and aggressive disease biology. Temporal changes in these features (delta radiomics) may further capture treatment-induced biological adaptation, including alterations in receptor expression or metabolic activity, thereby providing insight into differential treatment response across ISUP risk groups and enhancing clinical credibility. Consistent with this interpretation, Nardone et al. systematically reviewed the utility of delta radiomics across multiple oncologic endpoints, highlighting its ability to capture biologically meaningful longitudinal changes [32]. In prostate cancer specifically, Midya et al. demonstrated associations between sequential MRI-derived radiomic variations and pathological progression [33], while Delgadillo et al. showed that delta modeling from CBCT images could predict symptom burden and toxicity [34]. In parallel, dosiomics-derived features provide an interpretable link between spatial dose heterogeneity and biological effectiveness, supporting biologically guided dose adaptation. Collectively, these findings suggest that interpretable delta-radiomics and dosiomics features may complement established clinical and pathological factors, including ISUP grading, while remaining hypothesis-generating pending prospective, multi-institutional validation.

Some limitations must be acknowledged. We acknowledge that variability in the interval between pre- and post-treatment PSMA-PET/CT could influence delta-radiomics features. Post-treatment imaging was performed at a mean of 10 months, reflecting routine clinical surveillance practice. This time point was selected to allow for the resolution of acute post-radiotherapy inflammatory changes and stabilization of PSA response kinetics, thereby improving the biological interpretability of persistent or residual PSMA uptake. In our cohort, imaging clustered within a relatively consistent surveillance window, limiting extreme heterogeneity. Nevertheless, scan timing was not explicitly modeled or normalized, and formal time-stratified sensitivity analyses were not performed. This represents a limitation, and future studies incorporating time-adjusted delta modeling would further strengthen robustness. The present study should be considered exploratory in nature, given its retrospective design, the relatively small, single-institution patient cohort, and the limited number of metastatic events observed. These factors may reduce statistical power, and the absence of external validation may potentially limit the generalizability of the findings. As PSMA-PET/CT has only recently been incorporated into routine clinical practice, the availability of longitudinal post-treatment imaging and extended follow-up remains limited, further restricting definitive conclusions regarding long-term outcomes. Consequently, the study can be considered a proof-of-concept, and the results should be interpreted as hypothesis-generating. Future multicenter, prospective studies with larger cohorts, harmonized imaging protocols, and IBSI-compliant radiomics pipelines are required to validate these observations and establish their integration into the clinical pipeline. Integration of delta radiomics and dosiomics with molecular and genomic data may further enhance biological interpretability and support precision-guided treatment adaptation in prostate cancer.

Besides these limitations, this study possesses several notable strengths. It is among the first to comprehensively evaluate the prognostic value of delta radiomics and dosiomics derived from PSMA-PET/CT imaging in patients with localized prostate cancer. The use of PSMA-PET imaging provides a biologically specific and highly sensitive modality to assess intratumoral heterogeneity and treatment response beyond conventional morphologic metrics. By combining temporal and spatial descriptors with pre-treatment radiomics and clinical parameters, the study offers a multidimensional characterization of tumor behavior, integrating biological adaptation and dose-distribution heterogeneity. The analytical pipeline included standardized preprocessing, unsupervised feature selection, and internal validation in independent test datasets, ensuring methodological rigor and reducing overfitting risk. Moreover, the use of MFS as the primary endpoint adds clinical relevance, aligning with contemporary outcome benchmarks in localized PCa trials.

The integration of delta radiomics and dosiomics into clinical workflows could facilitate early identification of non-responders and support adaptive treatment strategies in localized prostate cancer. Temporal PSMA-PET changes may help personalize ADT or RT intensification, while spatial dosiomics features could inform biologically guided dose painting to optimize tumor control without increasing toxicity. As prospective data and multicenter collaborations mature, incorporating these imaging biomarkers into decision-support systems could refine patient selection for systemic therapy escalation or de-escalation. Ultimately, combining PSMA PET-derived radiomics, dosiomics, and clinical factors represents a step toward truly personalized RT, aligning biological response assessment with precision-based treatment planning in PCa.

## 5. Conclusions

This study demonstrates that integrating delta-radiomics and dosiomics features with pre-treatment PSMA-PET/CT radiomics and clinical variables significantly enhances the prediction of MFS in patients with localized PCa. By capturing both temporal biological changes and spatial dose heterogeneity, these spatiotemporal imaging biomarkers provide complementary prognostic information beyond conventional clinical parameters. The findings support the potential of PSMA-PET-based radiomics and dosiomics as non-invasive tools for personalized risk assessment, enabling more precise patient selection for treatment intensification or adaptation. Future prospective, multi-institutional studies with standardized imaging protocols are warranted to validate these results and advance the integration of such biomarkers into clinical RT practice.

## Figures and Tables

**Table 1:** Cox regression analysis results for delta-radiomics and dosiomics models showing concordance indices (c-scores) with 95% confidence intervals for training and test cohorts.

Model	Description	Train (c-Score, 95% CI)	Test (c-Score, 95% CI)
Delta-radiomics analysis			
Model 1_delta	Delta + pre-treatment + clinical	0.64 [0.58–0.65]	0.58 [0.53–0.59]
Model 2	Pre-treatment + clinical	0.62 [0.57–0.63]	0.56 [0.52–0.58]
Model 3	Clinical	0.57 [0.51–0.58]	0.51 [0.50–0.55]
Dosiomics analysis			
Model 1_dose	Dosiomics + pre-treatment + clinical	0.60 [0.56–0.61]	0.56 [0.54–0.59]
Model 2	Pre-treatment + clinical	0.58 [0.55–0.60]	0.55 [0.52–0.57]
Model 3	Clinical	0.55 [0.51–0.56]	0.50 [0.50–0.55]

**Table 2:** Five-year metastasis-free survival (MFS) binary classification results for delta-radiomics and dosiomics models, including sensitivity, specificity, and area under the curve (AUC) for both training and test datasets.

Model	Description	Train (Sensitivity, Specificity, AUC)	Test (Sensitivity, Specificity, AUC)
Delta-radiomics analysis			
Model 1_delta	Delta + pre-treatment radiomics + clinical	70.1%, 78.5%, 0.76	65.1%, 71.2%, 0.70
Model 2	Pre-treatment radiomics + clinical	65.2%, 70.3%, 0.71	58.2%, 63.5%, 0.65
Model 3	Clinical	56.4%, 65.6%, 0.63	52.1%, 60.4%, 0.56
Dosiomics analysis			
Model 1_dose	Dosiomics + pre-treatment radiomics + clinical	67.5%, 75.2%, 0.73	62.4%, 68.5%, 0.67
Model 2	Pre-treatment radiomics + clinical	63.4%, 68.1%, 0.68	55.1%, 60.2%, 0.62
Model 3	Clinical	54.1%, 62.4%, 0.60	51.3%, 57.1%, 0.54

## Data Availability

The workflow, parameters and algorithms used for analysis of the datasets used in the study are available from the corresponding author on reasonable request.

## Supplementary Materials

The following supporting information can be downloaded at: https://www.mdpi.com/article/10.3390/cancers18040677/s1, Table S1: A list of the radiomic features (107) extracted using PyRadiomics. The column headings indicate the family to which the list of features belongs to; Table S2: List of top eight features (delta-radiomics analysis) and top seven features (dosiomics analysis) selected through variance thresholding; Table S3: Cox-regression analysis results for models 1, 2 and 3 using feature set transformation with Principal Components (delta-radiomics analysis); Table S4: Five-year MFS binary classification results for models 1, 2 and 3 using feature set transformation with Principal Components (delta-radiomics analysis); Table S5: Cox-regression analysis results for models 1, 2 and 3 using feature set transformation with Principal Components (dosiomics analysis); Table S6: Five-year MFS binary classification results for models 1, 2 and 3 using feature set transformation with Principal Components (dosiomics analysis).
