# Supplementary material for: Prediction of Metastasis-Free Survival in Patients with Localized Prostate Adenocarcinoma Using Delta Radiomics from Pre-Treatment PSMA-PET/CT Scans and Dosiomics
Source: Cancers (Basel). 2026 Feb 19;18(4):677. doi: 10.3390/cancers18040677 (PMC12939975; doi:10.3390/cancers18040677)
Supplement: Supplementary file 1 [file cancers-18-00677-s001.zip › cancers-4115499-supplementary.pdf]

cancers

MDPI

Supplementary Materials: Prediction of Metastasis-free Survival in Patients with Localized Prostate Adenocarcinoma using Delta Radiomics from Pre-Treatment PSMA-PET/CT Scans and Dosiomics.

Apurva Singh, William Silva Mendes, Sang-Bo Oh, Ozan Cem Guler, Aysenur Elmali, Birhan Demirhan, Amit Sawant, Phuoc Tran , Cem Onal and Lei Ren

Table S1. A list of the radiomic features (107) extracted using PyRadiomics. The column headings indicate the family to which the list of features belongs to.

Shape	FirstOrder	GLCM	GLDM	GLRLM	GLSZM	NGTDM
Elongation	10Percentile	Autocorrelation	Dependence Entropy	GrayLevelNon Uniformity	GrayLevelNonU-niformity	Busyness
Flatness	90Percentile	Cluster Prominence	Dependence NonUniformity	GrayLevelNon Uniformity Normalized	GrayLevelNonU-niformity Normalized	Coarseness
LeastAxis Length	Energy	ClusterShade	Dependence NonUniformity Normalized	HighGrayLevel RunEmphasis	GrayLevel Variance	Complexity
MajorAxis Length	Entropy	Cluster Tendency	Dependence Variance	LongRun Emphasis	HighGrayLevel-Zone Emphasis	Contrast
Maximum2D Diameter Column	InterQuar-tileRange	Contrast	HighGrayLevel Emphasis	LongRunHigh GrayLevel Emphasis	LargeArea Emphasis	Strength
Maximum2D Diameter Row	Kurtosis	Correlation	Large Dependence Emphasis	LongRunLow GrayLevelRun Emphasis	LargeArea HighGrayLev-elEmphasis	
Maximum2D Diameter Slice	Maximum	Difference Average	Large Dependence HighGrayLevel Emphasis	LowGrayLevel RunEmphasis	LargeAreaLow-GrayLevel Emphasis	
Maximum3D Diameter	Mean Absolute Deviation	Difference Entropy	Large Dependence LowGrayLevel Emphasis	RunEntropy	LowGrayLevel-Zone Emphasis	
Mesh Volume	Mean	Difference Variance	LowGrayLevel Emphasis	RunLengthNon Uniformity	SizeZoneNonUni-formity	
MinorAxis length	Median	Id	Small Dependence Emphasis	RunLengthNon Uniformity Normalized	SizeZoneNonUni-formity Normalized	
Sphericity	Minimum	Idm	Small Dependence HighGray LevelEmphasis	RunPercentage	SmallArea Emphasis	
Surface Area	Range	Idmn	Small Dependence LowGrayLevel Emphasis	RunVariance	SmallArea HighGrayLev-elEmphasis	

 https://doi.org/10.3390/xxxxx

Surface Volume Ratio	RobustMean Absolute Deviation	Idn	ShortRun Emphasis	SmallAreaLow-GrayLevel Emphasis
Voxel Volume	RootMean Squared	Imc1	ShortRunHigh GrayLevel Emphasis	ZoneEntropy
	Skewness	Imc2	ShortRunLow GrayLevel Emphasis	Zone Percentage
	TotalEnergy	Inverse Variance		ZoneVariance
	Uniformity	JointAverage		
	Variance	JointEnergy		
		MCC		
		Maximum Probability		
		SumAverage		
		SumEntropy		
		SumSquares		

Table S2. List of top eight features (delta radiomics analysis) and top seven features (dosiomics analysis) selected through variance thresholding.

Delta radiomics analysis features	Dosiomics analysis features
DeltaCT_glrlm_RunEntropy	Dose_glcm_Autocorrelation
DeltaCT_firstorder_RobustMeanAbsoluteDeviation	Dose_firstorder_Entropy
DeltaPT_shape_Maximum3DDiameter	Dose_shape_Sphericity
DeltaPT_shape_Sphericity	Dose_glcm_SumSquares
PreCT_glrlm_RunLengthNonUniformityNormalized	PreCT_glrlm_GrayLevelNonUniformity
PreCT_glrlm_RunPercentage	PrePT_glrlm_ShortRunEmphasis
PrePT_firstorder_MeanAbsoluteDeviation	PSA_relapse
PSA_relapse	

Table S3. Cox-regression analysis results for models 1, 2 and 3 using feature set transformation with Principal Components (delta radiomics analysis).

Model	Train (c-scores, 95% CI)	Test (c-scores, 95% CI)
Model1_delta (delta+ pre+clinical)	0.61 [0.56, 0.62]	0.55 [0.52, 0.57]
Model2 (pre+clinical)	0.59 [0.54, 0.60]	0.53 [0.51, 0.56]
Model3 (clinical)	0.57 [0.51, 0.58]	0.51 [0.50, 0.55]

Table S4. Five-year MFS binary classification results for models 1, 2 and 3 using feature set transformation with Principal Components (delta radiomics analysis).

Model	Train [Sensitivity, Specificity, AUC]	Test [Sensitivity, Specificity, AUC]
Model1_delta (delta+pre+clinical)	[66.2%, 74.8%, 0.72]	[61.8%, 67.4%, 0.66]
Model2 (pre+clinical)	[62.7%, 67.2%, 0.67]	[54.6%, 59.7%, 0.61]
Model3 (clinical)	[53.8%, 61.7%, 0.59]	[50.8%, 56.5%, 0.54]

Table S5. Cox-regression analysis results for models 1, 2 and 3 using feature set transformation with Principal Components (dosiomics analysis).

Model	Train (c-scores, 95% CI)	Test (c-scores, 95% CI)
Model1_dose (dose+pre+clinical)	0.57 [0.54, 0.59]	0.54 [0.52, 0.56]
Model2 (pre+clinical)	0.56 [0.52, 0.57]	0.53 [0.51, 0.55]
Model3 (clinical)	0.55 [0.51, 0.56]	0.50 [0.50, 0.54]

Table S6. Five-year MFS binary classification results for models 1, 2 and 3 using feature set transformation with Principal Components (dosiomics analysis).

Model	Train [Sensitivity, Specificity, AUC]	Test [Sensitivity, Specificity, AUC]
Model1_dose (dose+pre+clinical)	[64.1%, 72.2%, 0.70]	[59.5%, 65.2%, 0.64]
Model2 (pre+clinical)	[60.4%, 65.5%, 0.65]	[52.7%, 57.3%, 0.59]
Model3 (clinical)	[51.6%, 60.1%, 0.56]	[50.0%, 54.6%, 0.53]
